# Analysis of Soil Structure Turnover with Garnet Particles and X-Ray Microtomography

**DOI:** 10.1371/journal.pone.0159948

**Published:** 2016-07-25

**Authors:** Steffen Schlüter, Hans-Jörg Vogel

**Affiliations:** 1 Dept. Soil Physics, Helmholtz-Centre for Environmental Research - UFZ, Halle, Germany; 2 Institut für Agrar- und Ernährungswissenschaften, Martin-Luther-Universität Halle-Wittenberg, Halle, Germany; Université de Technologie de Compiègne, FRANCE

## Abstract

Matter turnover in soil is tightly linked to soil structure which governs the heterogeneous distribution of habitats, reaction sites and pathways in soil. Thereby, the temporal dynamics of soil structure alteration is deemed to be important for essential ecosystem functions of soil but very little is known about it. A major reason for this knowledge gap is the lack of methods to study soil structure turnover directly at microscopic scales. Here we devise a conceptual approach and an image processing workflow to study soil structure turnover by labeling some initial state of soil structure with small garnet particles and tracking their fate with X-ray microtomography. The particles adhere to aggregate boundaries at the beginning of the experiment but gradually change their position relative to the nearest pore as structure formation progresses and pores are destructed or newly formed. A new metric based on the contact distances between particles and pores is proposed that allows for a direct quantification of soil structure turnover rates. The methodology is tested for a case study about soil compaction of a silty loam soil during stepwise increase of bulk density (*ρ* = {1.1, 1.3, 1.5} g/cm^3^). We demonstrate that the analysis of mean contact distances provides genuinely new insights about changing diffusion pathways that cannot be inferred neither from conventional pore space attributes (porosity, mean pore size, pore connectivity) nor from deformation analysis with digital image correlation. This structure labeling approach to quantify soil structure turnover provides a direct analogy to stable isotope labeling for the analysis of matter turnover and can be readily combined with each other.

## Introduction

Soil structure provides the pathways for matter fluxes, entails a high diversity of microhabitats and causes a heterogeneous distribution of reaction sites in soil. Through these regulatory traits it acts as a major driver for important soil functions like stabilization of soil organic matter, maintenance of biodiversity or water and nutrient cycling [1, 2]. Soil structure is not static, but continuously altered through abiotic (e.g. tillage, moisture changes) and biotic agents (e.g. bioturbation, root growth) [3]. These soil structure dynamics are also sometimes referred to as aggregate turnover, i.e. the continuous formation and destruction of aggregates especially in soils under agricultural use [4, 5].

Aggregate turnover rates are hard to quantify directly. However, the lifetime of organic matter (OM) in aggregates is strongly correlated with turnover of aggregates themselves [6]. Therefore stable isotope methods are sometimes applied to study soil structure dynamics indirectly. Labeled organic matter enriched in ^13^C or ^15^N [7–9] or a change in cultivation from C3 to C4 plants with natural differences in C isotope ratios [4, 10] are used to study the fate of OM in soil (for comprehensive reviews see [3, 11, 12]). After a certain incubation time the soil is discerned into different aggregate size classes through wet sieving and OM turnover rates are derived from the proportion of labeled OM in each aggregate size class. This approach to linking OM turnover to structure turnover is often motivated by the aggregate hierarchy concept first proposed by Tisdall &*amp*; Oades [13]. In this conceptual framework, soil structure is organized in different levels ranging from organo-mineral complexes, to microaggregates (< 250*μ*m) and macroaggregates (> 250*μ*m). The predominant binding agents at each level differ in lifetime from very stable to transient. Macroaggregates are deemed to be formed and destructed relatively fast, whereas microaggregates are formed slowly within macroaggregates and are the main driver for OM stabilization in soil. An indirect inference about structure turnover from fractions of labeled OM in different aggregate size pools can be made under the assumption that macroaggregate formation and microaggregate formation rates are the ultimate cause for the observed fractionation of labeled OM. However, the validity of this aggregate hierarchy concept and therewith the congruency of OM turnover and aggregate turnover seems to be limited to soils rich in clay and silt and rather high OM concentrations [14]. Moreover, labeled OM might be stabilized by other mechanisms than physical protection in microaggregates, such as inherent recalcitrance, association with minerals, or a shortage in readily available organic compounds required as an energy source for microbial decomposition of labeled OM [15, 16].

A similar approach to estimation of aggregate turnover rates is provided by studying the fate of microparticles, e.g. ceramic dysprosium oxide tracer spheres (d: 0.4 mm) that can be detected through neutron emission [5]. These spheres were added to a field soil with a rototiller and from their recovery in different aggregate size classes after wet sieving a macroaggregation rate was derived. This derivation of aggregate turnover rates from aggregate stability during wet sieving has obvious flaws. Aggregation or more precisely the disintegration of aggregates strongly depends on the energy input and forces that act on them during wet sieving [17]. Comparability among different studies therefore requires highly standardized laboratory protocols. It does, however, not imply the existence of clearly separable aggregates within undisturbed soil [18]. Instead, soil structure manifests itself through a complex network of pores at all scales and their heterogeneous distribution in space evokes preferential failure zones during mechanical disturbance. This complex patterns of pores ultimately governs the distribution of water, nutrient supply and oxygen levels and should therefore be studied in its original context. In summary, indirect methods to quantify structure turnover rates are flawed because measures based on OM turnover and aggregate stability are not necessarily correlated with the formation and destruction of pores at microscopic scales.

Imaging techniques like X-ray microtomography (*μ*CT), in turn, provide a detailed view into the physical structure of undisturbed soil at a spatial resolution of a few microns. The three-dimensional images do not only enable a visual inspection of the internal structure of opaque soil but are also amenable to quantitative image analysis. The spatial attributes which can be derived from *μ*CT images are numerous and range from pore size distribution and pore connectivity [19], to spatial correlation of pores [20] and distances between pores and occluded particulate OM [21, 22], just to name a few. Repeated sampling of field soils during a growing season facilitates the detection of statistical changes in pore space attributes, e.g. during a growing season [23, 24]. Incubation experiments in the laboratory allow for a more detailed look on soil structure dynamics, since the same samples can be scanned repeatedly under controlled conditions [25–27]. This allows for a direct analysis of changing pore size distributions or pore connectivities as a function of e.g. different C input or different microbial activity. Yet, this is still a statistical evaluation of pore space changes and therefore only provides indirect clues on structure turnover. A rather new method to directly study the movement of soil constituents during soil structure development is called digital image correlation (or digital volume correlation) [28–30]. The rationale of this method is to recover the deformation field in soil by image registration. That is, the image of a deformed soil is aligned to an image of the original soil and the transformation matrix that resulted in an optimal spatial alignment is used to calculate the deformation field. This yields detailed patterns of how much soil has been displaced how far in which direction. Digital image correlation and related methods like particle image velocimetry have mainly been used to study soil deformation through mechanical stresses and its great potential to investigate structure turnover is not yet explored. One major hurdle for a direct assessment of structure dynamics is that the displacement of soil constituents needs to be studied in its spatial context. For instance, OM located at the surface of a macropore may end up in the center of a newly formed aggregate, if the macropore is closed due to compression, even though the active displacement of this OM may have been negligible. Likewise, crack formation due to drying may expose formerly occluded OM and create a completely new microenvironment without any OM translocation in space.

In this paper we present a new image processing protocol that allows for a direct quantification of structure dynamics by tracking strategically positioned microparticles. The chosen garnet particles contain iron oxide with high X-ray attenuation so that they can be easily detected in spite of their small diameter of a few voxels (d: 0.045–0.1 mm). Aggregate packings are prepared from sieved aggregates covered with microparticles. In this way, the position of garnet particles marks the delineation of initial macroaggregates. The biotic and abiotic drivers of soil structure changes operate on very different time scales and not all of them can be captured in a meaningful way by laboratory experiments. In this study we induce structure changes exemplarily through soil compaction, which typically occurs in agricultural soils under tillage. The position of individual particles at different levels of soil compaction is evaluated with respect to distances to the nearest pore. These new metrics are underpinned with conventional pore space attributes and metrics derived from digital image correlation to provide a sound picture of soil structure changes at a spatial scale of a few microns. Finally, the findings for structure dynamics during compaction are used to conceptualize an approach to quantify soil structure turnover rates.

## Materials and Methods

Soil was collected from the upper 5 cm of a Haplic Chernozem (WRB classification) developed from loess and managed as bare fallow at the Experimental station (51.3943*N*, 11.8777*E*) of the Helmholtz-Centre for Environmental Research—UFZ in Bad Lauchstädt, Germany. The owner of the land gave permission to conduct the study on this site. Soil texture was composed of 11% sand, 68% silt and 21% clay (Sedimat 4–12, UGT GmbH, Müncheberg, Germany). The organic carbon and total nitrogen content of the soil were C_*org*_ = 2.05% and N_*t*_ = 0.19%, respectively (Elementar Analysator Vario EL cube, Elemantar Analysensysteme GmbH, Hanau Germany). The fresh soil was sieved under moist conditions close to field capacity. Aggregates in the size range of 0.5–2 mm were collected and stored in the fridge at 4°C. Weeks later the aggregates were carefully moistened by putting them on a moist paper towel for 30 min, so that the gravimetric water content in the aggregates adapted to 10.5±1.3%. Subsequently, the aggregates were fully covered with garnet fine sand (Garnit #240, Kuhmichel Abrasiv GmbH, Ballenstedt, Germany) with a grain size of 45–100 *μ*m. This garnet fine sand, from here on denoted as particles, consists of the mineral Almandine, which is composed of 33% Fe_2_O_3_. This iron content leads to a better contrast against the surrounding soil than conventional quartz sand [31], but does not lead to a drastic attenuation of X-ray photons like similar sized particles of metallic iron so that shading artifacts are avoided. The aggregates were gently shaken on a 0.2 mm sieve for 1 min to remove excess particles. 5 g of these moist aggregates were filled into 5 ml plastic syringes with an inner diameter of 12.5 mm (5 replicates). Occasionally some coarse garnet grains (Garnit #12-20, Kuhmichel Abrasiv GmbH, Ballenstedt, Germany) with a grain size of 0.5–1.2 mm were added to the aggregate packing. These coarse garnet grains, from here on denoted as grains, are easily detected in the CT images and serve as additional position markers for subsequent image analysis. The dry weight of this packing of moist aggregates was determined from equally prepared samples (3 replicates) after oven drying at 105°C for 24 h. Bulk density was then adjusted in steps (1.1 g/cm^3^, 1.3 g/cm^3^, 1.5 g/cm^3^) by compressing the soil with the piston of the syringe to a specific volume. This uni-axial, static compression is likely to cause qualitatively different compaction than elastic soil displacement through dynamic loading during wheel traffic. Nevertheless it fulfills the purpose to evoke some easily reproducible changes to the soil structure on which our new conceptual approach can be tested.

After each compression step the samples were scanned with X-ray microtomography (X-TEk XCT 225, Nikon Metrology). The energy settings (80 keV, 120 *μ*A, no filter) resulted in good contrast without overexposure at the lateral margins of the detector panel. An entire scan comprised 2300 projections with an exposure time of 1 s (one frame per projection). The syringes were scanned in two-heights to cover ≈ 60% of the total height of the uncompressed aggregate packing. The reconstruction of three-dimensional images via filtered back projection was done with the CT Pro 3D software package (version 3.1) at a spatial resolution of 8*μ*m and 8-bit gray scale resolution.

**Fig 1:**
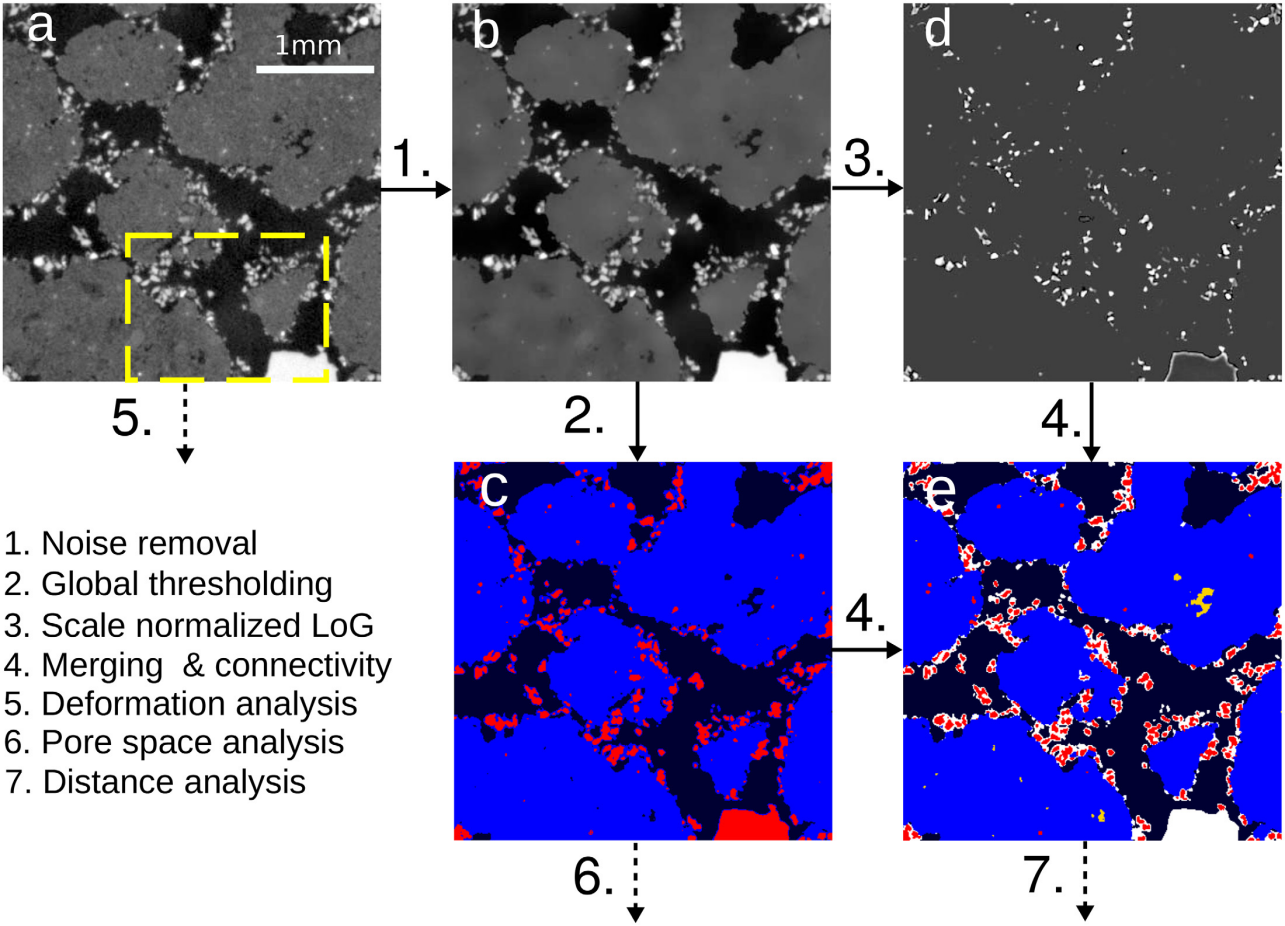
Image processing workflow for this study depicted for a small two-dimensional subset (a). Noise is removed with a non-local means filter (b). Image segmentation is performed in several steps. First, gray values are tentatively segmented into pores (black), aggregates (blue) and garnet (red) via simple thresholding (c). Then particles are detected with a Laplacian of Gaussian Filter (LoG) (c) and subsequent hysteresis thresholding of the LoG Image. Note that the edges of large grains are masked out during particle detection (not shown). For the final segmentation (e) partial volume voxels tentatively assigned to the aggregate class are set to unassigned (white) with a morphological opening of the aggregate class by a small structuring element (*d*_*SE*_ = 5 voxels). The tentative garnet class is set to unassigned (white) and overwritten by the thresholded LoG image (red). Pores are further differentiated with respect to whether they are fully enclosed in soil aggregates (yellow) or not (black) (f). These images are subjected to different types of analysis (5.-7.).

The raw images are subjected to an image processing workflow that was especially tailored for this study (Fig 1). The entire workflow is described in detail in S1 File by means of the small subset within the yellow frame in Fig 1(a) and only briefly summarized in the following. Noise in the raw images was removed with a non-local means denoising filter (Fig 1b) [32]. Thresholds for three classes are detected automatically according to [33]. Global thresholding leads to a tentative segmentation into pores (black), aggregates (blue) and garnet (red) (Fig 1c). Partial volume effects due to limited image resolution cause false aggregate voxels around particles. These thin films of aggregate voxels around particles are removed by a combination of erosion, removal of small objects and dilation. The identification of particles is improved with a Laplacian of Gaussian (LoG) filter [34] for blob detection (Fig 1d). The garnet particles are segmented with hysteresis thresholding of the scale normalized LoG result [33, 35]. The combination of blob detection and partial volume voxel treatment leads to an improved segmentation result (Fig 1e). The tentative pore class is labeled differently depending on whether pores are fully enclosed by aggregates (yellow) or connected to the interaggregate pore space (black). All removed aggregate and garnet voxels are gathered in an unassigned class (white).

Quantitative analysis of the CT images is directed towards different ends (step 5.-7. in Fig 1):

- The deformation analysis via digital volume correlation is performed on the raw images. The workflow for digital image correlation is described in [30] and implemented in elastix [36]. As preprocessing steps the image dimensions are rescaled by a factor of four in each direction to reduce computational costs and the grayscale is rescaled such that all pores and aggregates are black and garnet grains are depicted with optimal contrast. It has been shown previously that this improves the image registration of the deformed grain matrix onto the original grain matrix [30].
- The pore space analysis is done for both the interaggregate pore space and isolated pores together. Depth-dependent changes in pore volume are detected with porosity profiles, i.e. the area fraction of pores in each xy-plane. Pore size distributions are computed with the maximum inscribed sphere method as implemented in the BoneJ plugin for ImageJ [37]. Pore connectivity *Γ* is computed from the size distribution of individual pore clusters [38]:
  $$\begin{array}{c} \Gamma =\frac{1}{N_{v}^{2}}\sum_{i=1}^{N_{c}}n_{i}^{2} \end{array}$$ (1)
  where *N*_*v*_ is the number of pore voxels, *N*_*c*_ is the number of individual pore clusters and *n*_*i*_ is the number of voxels in cluster *i*. This second moment of the cluster size distribution equals one if all pores are connected in one percolating cluster and converges to zero if porosity is fragmented into many clusters of similar size.
- Distance analysis: A Euclidean distance transform as implemented in the 3D Image suite for ImageJ [39] determines the minimum distance of each voxel (including aggregates, garnet and occluded pores) to the interaggregate pore space. This is also referred to as the contact distribution [40]. The average of this contact distribution is an estimate for the mean diffusion lengths of air into the soil matrix assuming that small unresolved pores remain water-filled. On top of that the Euclidean distances between garnet particles and air-filled pores can be determined and compared to that of any point within the soil matrix.

## Results

### Porosity changes due to compaction

**Fig 2:**
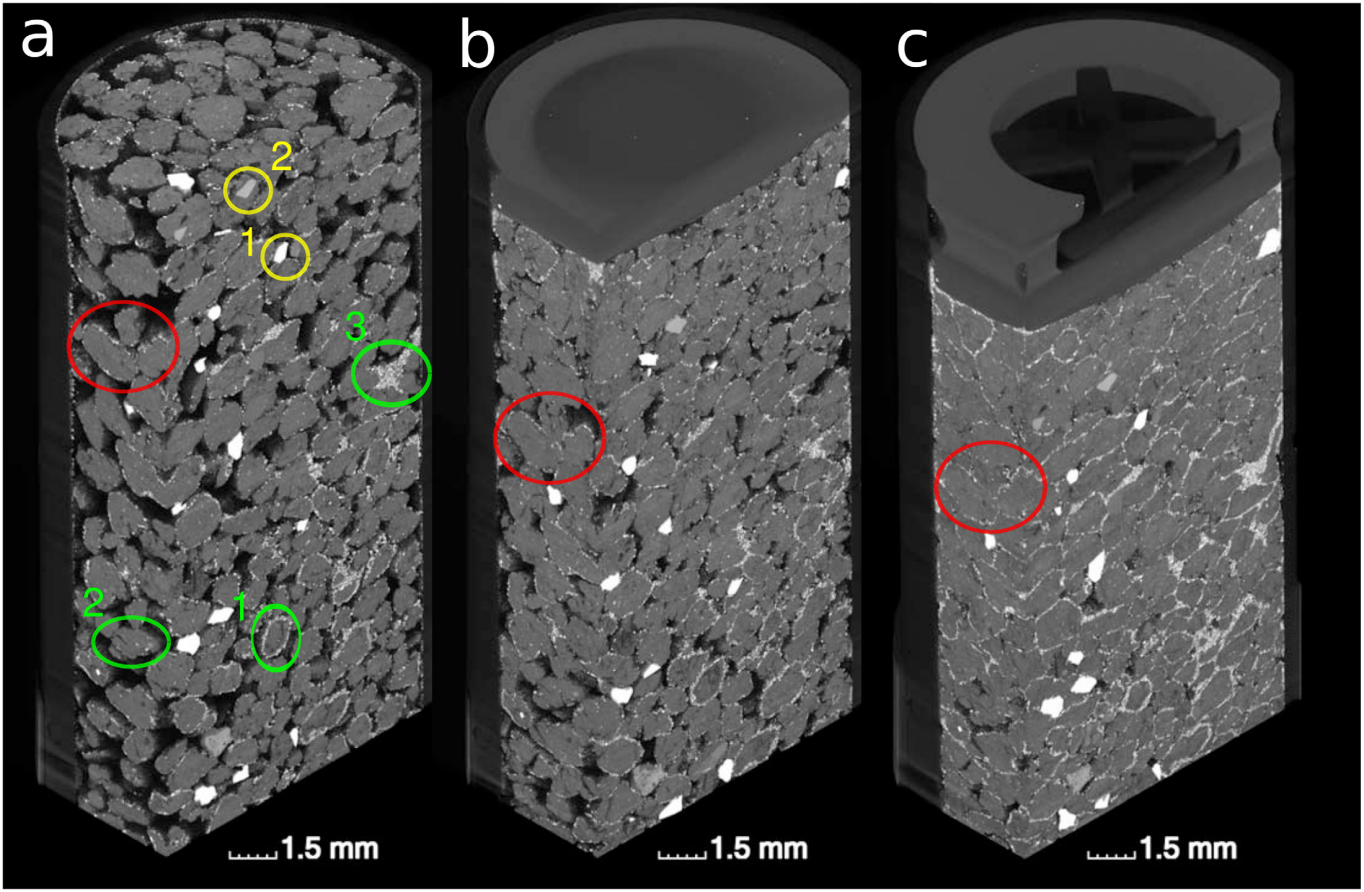
3D rendering of a sample at a bulk density of (a) *ρ* = 1.1 g/cm^3^, (b) *ρ* = 1.3 g/cm^3^ and (c) *ρ* = 1.5 g/cm^3^. The green circles highlight aggregates which are strongly (#1) or weakly (#2) covered with particles or pores in which detached particles gather (#3). The yellow circles highlight that photon absorption in garnet (#1) is higher than in iron-free minerals like quartz (#2). The red circles highlight in the incorporation of particles into the soil matrix in the course of compaction.

The changes in soil structure due to compaction are depicted in (Fig 2). The uncompacted soil at a bulk density of *ρ* = 1.1 g/cm^3^ exhibits a loose aggregate packing. The coverage of aggregates with garnet particles is not perfectly homogeneous (Fig 2a, green circles). Some aggregates, probably wetter than others during sieving, are covered with a thick layer of particles (green #1), while other aggregates are only sparsely covered with particles (green #2). Upon drying and/or unintentional shaking some particles detach from the aggregates and gather at pore constrictions (green #3). Bigger garnet grains are randomly distributed across the sample. This grain matrix will be the basis for subsequent deformation analysis. The different X-ray adsorption of garnet (yellow #1) and grains of iron-free minerals like quartz (yellow #2) is clearly visible. The piston of the syringe enters the field of view from above during uni-axial compression of the soil to a bulk density of *ρ* = 1.3 g/cm^3^ and *ρ* = 1.5 g/cm^3^ (Fig 2b and 2c). Soil compaction does not occur uniformly. Pores in front of the piston are strongly compressed, whereas the pore space further away from the piston is less affected. The position of garnet particles in relation to macropores changes drastically through soil compaction (red boxes). In uncompacted soil almost all garnet particles are in direct contact with air due to the way the sample was prepared. At an intermediate bulk density a large fraction of particles is already occluded between aggregates. At the highest bulk density many pores vanished completely so that the former aggregate boundaries can only be identified through linings of garnet particles.

**Fig 3:**
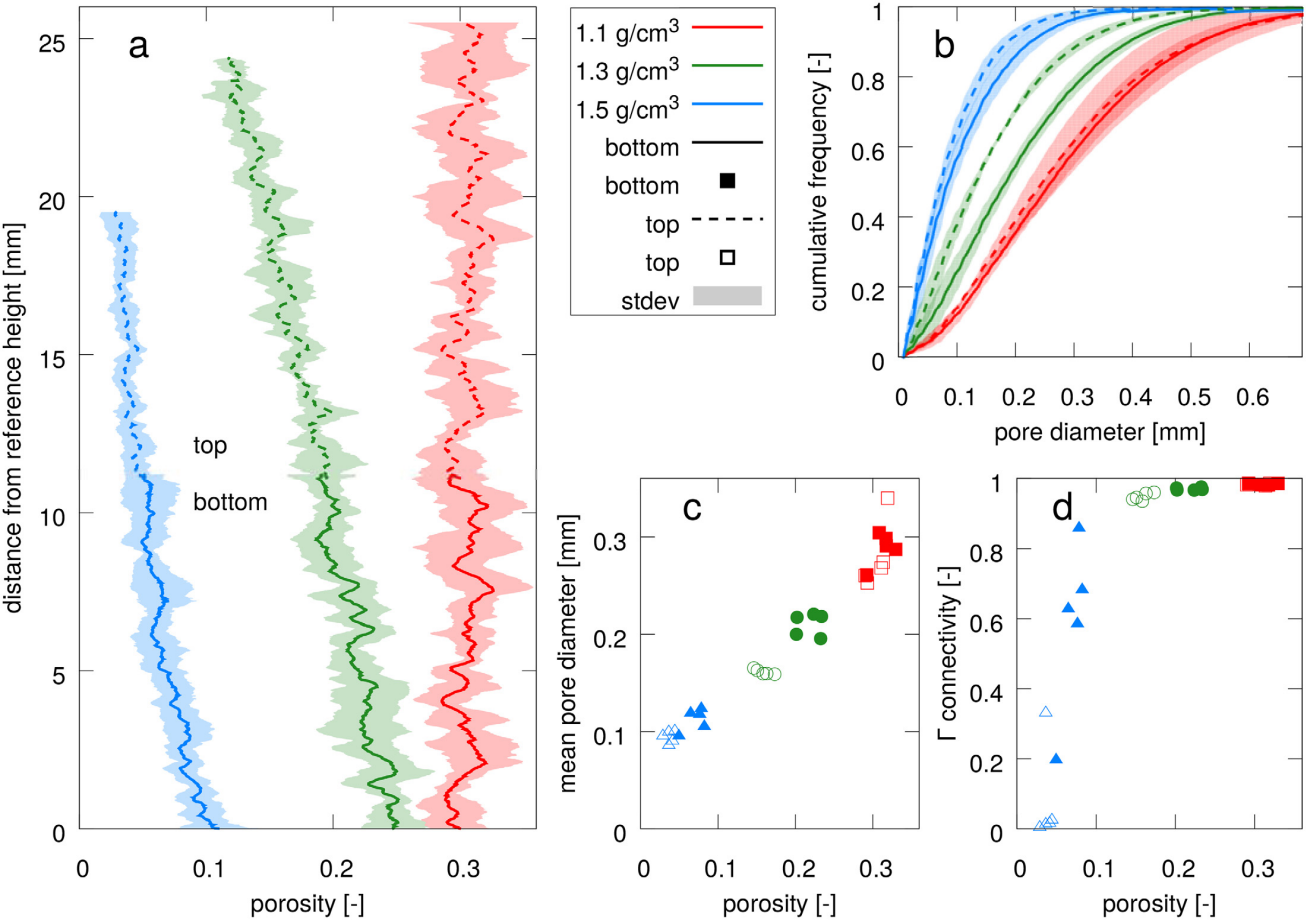
Pore space analysis for soil at three bulk density levels (*ρ* = 1.1, 1.3, 1.5 g/cm^3^) with five replicates: (a) porosity profiles, (b) cumulative pore size distribution, (c) mean pore size and Γ connectivity as a function of pore diameter. Because of the depth gradient in porosity results are shown separately for the top and bottom of the samples.

In the following these visual observations will be confirmed by quantitative image analysis. First, the different bulk densities are compared with respect to height profiles of porosity (Fig 3a). Sample preparation at the lowest bulk density (*ρ* = 1.1 g/cm^3^) lead to a uniform porosity profile around an average porosity of *ϕ* = 0.30. The uni-axial compression to a bulk density of *ρ* = 1.3 g/cm^3^ caused a linear decrease in porosity from *ϕ* = 0.24 at the bottom of the field of view to *ϕ* = 0.12 at the top in close proximity to the piston (average *ϕ* = 0.18). The second compaction to a bulk density of *ρ* = 1.5 g/cm^3^ reduced average porosity further to an average of *ϕ* = 0.05, again showing a linear decrease in porosity from the bottom of the field of view to a height of about 15 mm. Above that height an irreducible porosity of *ϕ* = 0.03 is reached.

Due to the porosity gradient in the sample all subsequent analysis will be presented separately for the top and bottom of the sample. The cumulative pore size distribution (Fig 3b) clearly shows a shift in the range of pore diameters with changing bulk density. An increase in bulk density leads to a shift in the pore size distribution towards smaller pore diameters. At the same time the curves become steeper, i.e. the range of prevalent pores diameters gets narrower as big pores get compacted more easily. Interestingly, the average pore diameter (Fig 3c) scales linearly with porosity. Note that this only refers to porosity above the resolution limit of 8 *μ*m. Connectivity, in turn, exhibits a very non-linear relationship with porosity. Above a critical porosity of 10–12% the pore space is well connected and only a small fraction of pores is not connected to the main, percolating pore cluster. Close to the percolation threshold a small reduction in porosity entails a huge fragmentation of the pore network.

### Soil deformation

**Fig 4:**
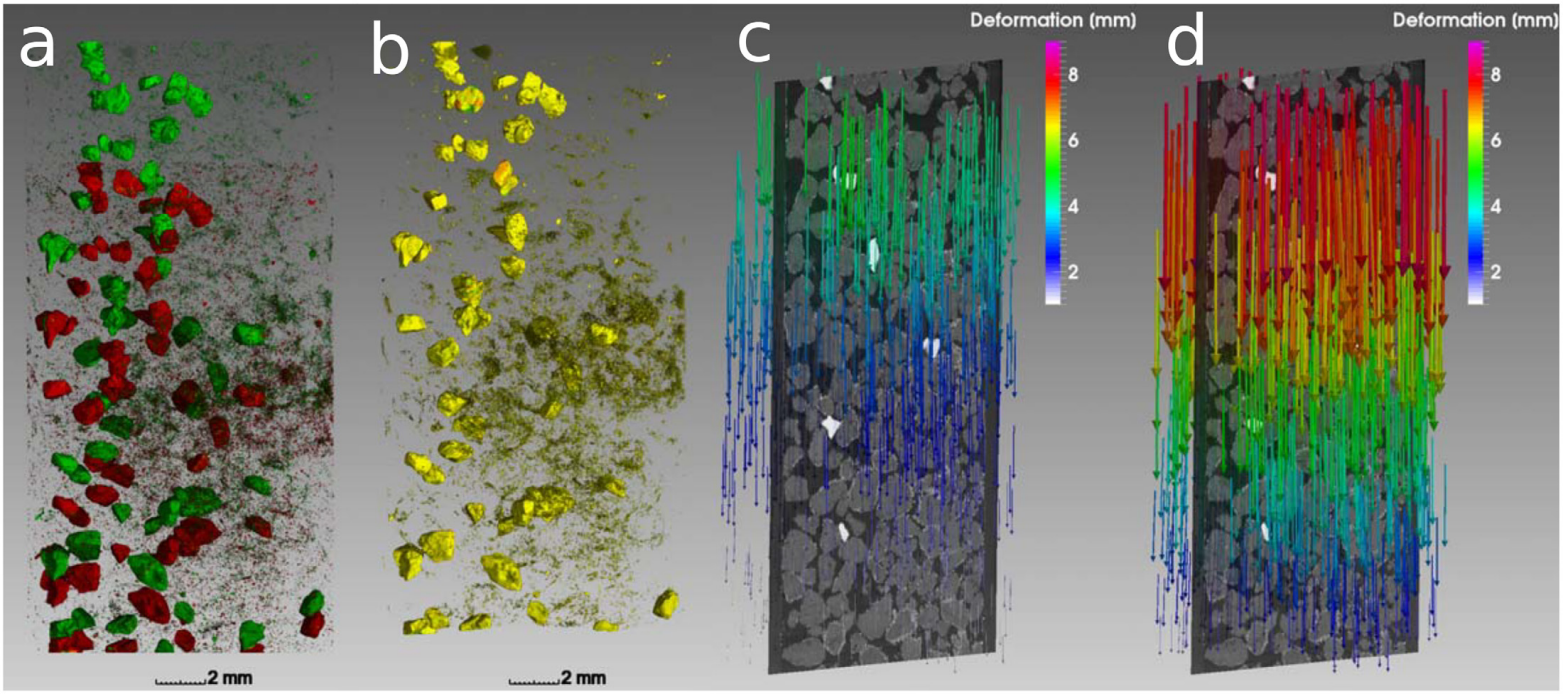
(a) Spatial distribution of big garnet grains and small garnet particles at a bulk density of 1.1 (green) and 1.3 g/cm^3^ (red). (b) Elastic image registration leads to a very good spatial alignment between the deformed and the original image. (c) The resulting displacement field shows a gradient in vertical displacement. (d) Compaction to 1.5 g/cm^3^ increases this gradient even further.

The deformation of soil during compaction is analyzed with digital image correlation. The displacement of individual garnet grains during compaction from *ρ* = 1.1 g/cm^3^ to *ρ* = 1.3 g/cm^3^ for one out of five replicates is depicted in Fig 4(a). Evidently the displacement is smaller at the bottom of the field of view and strongest close to the piston of the syringe. The exact displacement of garnet grains is computed with elastic registration of the deformed image onto the original image using a B-spline transform of a regular grid of control points. A successful registration is achieved for all grains and even for larger clusters of particles, as indicated with yellow color in Fig 4(b). The resulting deformation field shows a gradual increase in compaction with vertical position ranging from 1.5 mm at the bottom to 5.5 mm at the top of the field of view (Fig 4c). The compaction to *ρ* = 1.5 g/cm^3^ increases the vertical displacement to 2.5 mm at the bottom and 8.5 mm at the top. (Fig 4(b)). There is only little lateral heterogeneity in the downward movement (z-components of the vectors) and hardly any horizontal movement of garnet grains (x-y components of the vectors).

### Particle distribution

**Fig 5:**
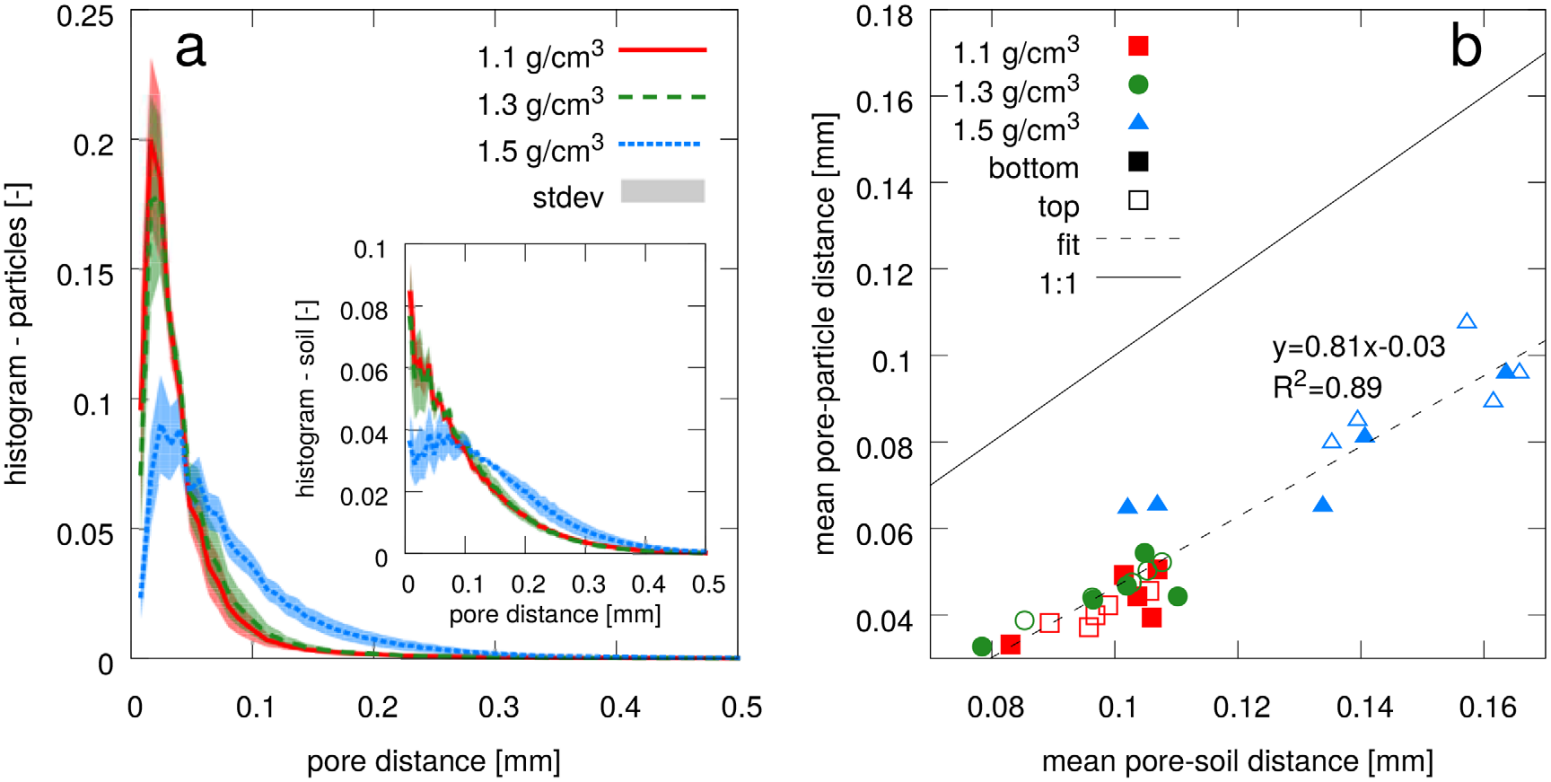
(a) Frequency distribution of contact distances between particles and pores with average and standard deviation of five replicates. The inset shows the contact distance distribution between bulk soil and pores with is generally much larger. (b) The mean contact distance of particles and bulk soil is shown for each replicate and further separated into top and bottom part of the sample. The mean contact distance for particles scales linearly with the mean contact distance for bulk soil during compaction.

Pores may disappear completely during soil compaction. As a consequence the minimum distance from any location within the soil matrix to the nearest pore undergoes characteristic changes. This is summarized in the histogram of contact distances for all non-pore voxels (Fig 5a, inset). The aggregate packing at *ρ* = 1.1 g/cm^3^ renders a lot of aggregate surfaces in direct vicinity to a well connected pore network. The exponential decline in frequency with increasing contact distance is a consequence of the compact shape of aggregates, which are all of similar size. When only the garnet particles are considered (Fig 5a), the decline is much steeper. Evidently, all garnet particles have only small contact distances at the beginning of the experiment, because they adhere to aggregate surfaces. The natural fine sand fraction of the soil, which is also detected as particles during image processing, only evokes a minor tailing of the histogram for distances above 0.1 mm. When the soil is compacted to *ρ* = 1.3 g/cm^3^ the frequency distribution of contact distances does not change, neither for garnet particles nor for all non-pore voxels in general. Only at a bulk density of *ρ* = 1.5 g/cm^3^ there is a considerable shift towards greater contact distances because a large fraction of garnet particles is not in direct contact with the pores anymore.

The comparison between average pore distance of soil and garnet particles (Fig 5b) exhibits the following features: (1) There is a linear relationship between average contact distance for particles and average contact distance for soil during soil compaction. (2). All data points are below the 1:1 line, i.e. on average the particles remain closer to pores as compared to bulk soil. (3) Differences in the pore space attributes of the top and bottom parts do not entail different contact distances.

## Discussion

### Relationship between contact distances and porosity

Bulk density changes due to soil compaction affect soil structure in many ways. Some pore space attributes like porosity and mean pore diameter scale linearly with bulk density. Pore connectivity, in turn, exhibits a very non-linear behavior. At first, compaction only leads to a reduction of pore sizes without a disruption of the pore network. Only when a critical porosity threshold of 10–12% is reached, the well-connected pore network breaks into many evenly-sized, isolated pores. The sharp transition in pore connectivity in a narrow porosity range is due to the regular packing of similar-sized aggregates. The transition is likely to be more gradual in natural soil with a more irregular network to start with.

**Fig 6:**
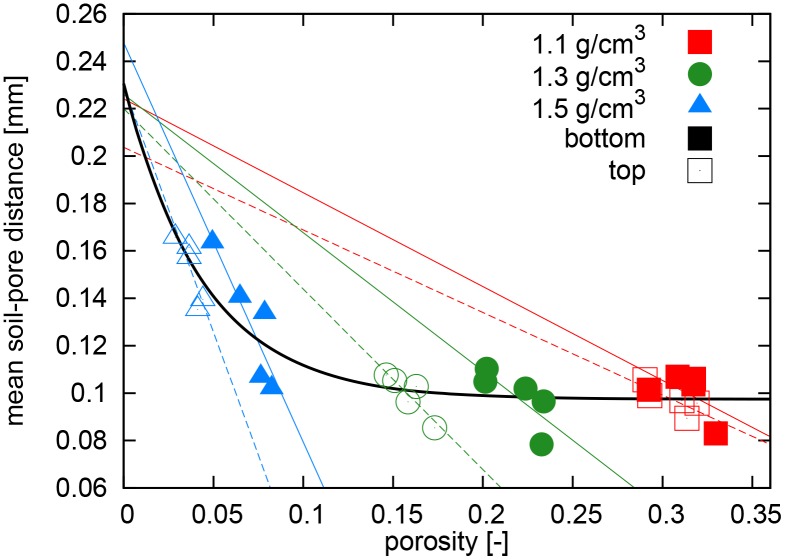
The mean contact distance between bulk soil and pores scales exponentially with decreasing porosity during soil compaction. This exponential trend follows from the non-linear increase of contact distances as pores start to vanish completely at higher bulk density. The scatter with subgroups of similar bulk density is caused by different degree of intra-aggregate porosity mostly due to crack-formation. This variability in intra-aggregate porosity causes a linear scaling relationship between contact distances and porosity. All fitted curve converge to a similar contact distance at vanishing porosity which is mainly determined by the average size of aggregates.

Looking at soil structure changes from the perspective of the pore space seems to be the natural choice, since many important soil functions like aeration, water storage or solute transport depend on the size distribution and continuity of pores. However, soil functions which relate structure-mediated accessibility of soil constituents calls for a shift of focus towards soil matrix attributes. For instance, the physical protection of particulate organic matter against microbial decomposition within soil aggregates is mainly governed by diffusion-limited supply of nutrients, oxygen and exoenzymes through predominantly water-filled intra-aggregate pores, which cannot be captured by the image resolution of 8*μ*m. For oxygen, this information on accessibility is best described by the diffusion length from the substrate through the soil matrix to the next air-filled pore [22, 41]. Evidently, this diffusion length is a transient property that depends on soil matric potential *ψ*_*m*_. At the image resolution of 8*μ*m used in this study we cover the pore space which is air-filled at a matric potential of *ψ*_*m*_ = −375 hPa (derived from Young-Laplace law assuming perfect wettability). If *ψ*_*m*_ was controlled during the experiment and air and water was segmented separately in the *μ*CT images or if the distribution of air and water was modelled e.g. with maximum inscribed sphere analysis of the pore space [42, 43], then the contact distances to air-filled pores, could be examined for any matric potential higher (i.e. moister) than *ψ*_*m*_ = -375 hPa. Hence, based on the contact distances, it is possible to evaluate diffusion lengths into water-filled regions for a wide moisture range as typical for many soils. This average contact distance undergoes non-linear changes with decreasing porosity caused by compaction (Fig 6). At *ρ* = 1.3 g/cm^3^, the reduction in porosity does not lead to increased contact distances, because most pores shrink in size, but not beyond the resolution limit. Only at a critical porosity of 10–12% there is a substantial occlusion of pores within the dense soil matrix or even a complete removal of resolved porosity. Note that this porosity threshold coincides with the steep decline in pore connectivity (Fig 3d). The black curve fitted to the data in Fig 6 suggests an exponential decline in mean contact distance with increasing porosity. In S2 File we show that this exponential trend is in line with contact distances for overlapping spheres of different packing geometry and a diameter comparable to the maximum inscribed sphere of the irregular-shaped aggregates used in this study. This indicates that the exponential trend is caused by changes in interaggregate porosity, i.e. by a change in void space between aggregates.

Interestingly, the scatter within the different compaction levels is quite high. However, this scatter is not random, but shows a negative linear trend between porosity and mean contact distance for each bulk density sub-group. At constant bulk density, an increased porosity is mainly due to additional intra-aggregate pores, which mainly results from micro-crack formation that reduces the mean contact distance to pores. All fitted curves converge towards the same mean contact distance for vanishing porosity, which we show in S2 File to mainly depend on the average size of aggregates.

In summary, this assessment of soil structural dynamics via changes in contact distances is a valuable information based on the spatial arrangement of the soil, which cannot be inferred from a statistical analysis of pore space attributes alone. By analyzing the distribution of water and air in the pore space, changing diffusion pathways due to water dynamics or soil structure dynamics could potentially be treated separately.

### Measuring soil structure turnover

In the following, we will therefore use contact distances to outline a direct approach to measuring soil structure turnover. To do so, we make use of a close analogy to stable isotope methods as a standard method to quantify turnover rates of different carbon pools in soil. By labeling of a certain pool or substrate its fate in the carbon cycle can be monitored over time and net fluxes can be derived even under steady-state conditions. Likewise, soil structure can be distinguished in two pools according to their spatial context: (i) regions in direct contact with oxygen, e.g. aggregate surfaces and the soil around macropores, and (ii) the interior of aggregates where OM is potentially protected from mineralization. Pulse labeling of the soil structure is then achieved by covering aggregate surfaces with inert particles and the fate of these particles can be studied over time.

**Fig 7:**
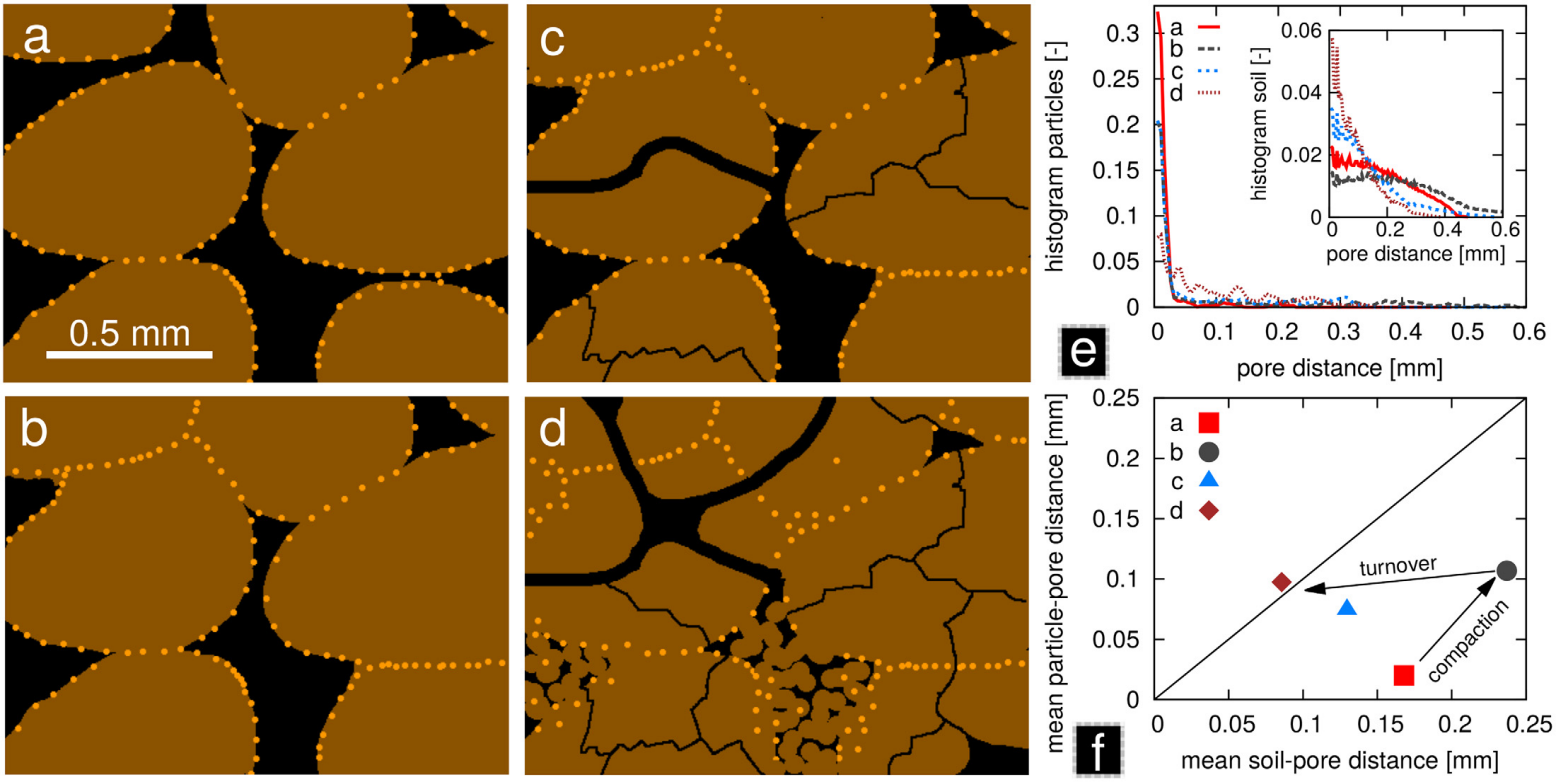
Conceptual scheme for the quantification of soil structure turnover rates. (a) A two-dimensional packing of aggregates is covered with garnet particles. (b) The aggregates are first compressed by soil compaction. Subsequently, soil structure turnover is initiated through new root channel formation, micro-crack formation and the partial refilling of pores with earthworm casts, where (c) and (d) represent two consecutive moments in time. (e) The distribution of contact distances between pores and particles or between pores and bulk soil (inset). (f) The mean contact distance of particles is initially much smaller than the mean contact distance for bulk soil. Soil compaction does not lead to a trajectory towards the 1:1 line (randomized position of particles), whereas structure turnover does.

This is illustrated in the conceptual scheme in Fig 7. If particles were distributed randomly across soil, then the mean contact distance between particles and pores should equal the mean contact distance between all soil voxels and pores. Evidently, the distribution of particles at the beginning of the structure labeling experiment is far from being random, as all particles adhere to the surface of aggregates (Fig 7a). That is to say, the pool of small contact distances is strongly enriched with particles. We have shown in our experiment, that soil compaction has only a small effect on the ratio between particle contact distances and soil contact distances (Fig 5) in spite of large physical displacement of soil constituents during compaction (Fig 4). Many particles become occluded within bigger aggregates so that their minimum distance to the next pore increases, but so does the pore distance for bulk soil in general (Fig 7b). As a consequence, the trajectory in Fig 7(f) proceeds in parallel to the 1:1 line. Actual soil structure turnover, i.e. the formation and destruction of pores through rearrangement of soil constituents by abiotic and biotic agents, will likely cause a different trajectory. This soil structure turnover is illustrated with different degrees of bioturbation (root channels, refilling of pores with earthworm casts) and micro-crack formation Fig 7(c) and 7(d). As a result, the mean contact distance of particles approaches that of bulk soil. The spatial distribution of particles may not be random and still demarcate former aggregate boundaries. However, it becomes statistically similar to the distance distribution of bulk soil as the trajectory approaches the 1:1 line in Fig 7(f). Soil structure has reached a dynamic equilibrium then, because continued turnover will only randomize the spatial distribution of particles even further without diverging from the 1:1 line. The rate at which this dynamic equilibrium is reached can be interpreted as the turnover rate of soil structure. Evidently this turnover rate will depend on the activity of biotic and abiotic agents, but also on whether old pores are continuously reused or newly formed and destructed. This effect of natural processes of structure formation on contact distances will be investigated in a future structure labeling study.

### Practical considerations

From a practical point of view, a structure labeling experiment will always entail some degree of initial disturbance to bring the garnet particles into contact with the soil matrix. We coated the surfaces of sieved aggregates with fine sand particles that are indistinguishable from the fine sand fraction of soil. Our study represents an extreme case of pulse labeling, since the soil matrix was almost devoid of fine sand and many garnet particles were used. Such a structure labeling approach would still be feasible with soils naturally containing a considerable fine sand fraction or lower coverage of aggregate surfaces with garnet particles, as long as the pool of short constact distances is significantly enriched after structure labeling. Moreover, packings of sieved aggregates respresent a rather artificial soil structure to begin a structure dynamics experiment with. In a more natural setting garnet particles could be added to a field soil during plowing or harrowing similar to [5] under the tacit assumption that particles are preferably located at short contact distances due to the way they are added during tillage. Evidently the total abundance of garnet particles would be much lower in this case and the imbalances of initial contact distances are less strong. In analogy to stable isotope methods this is more similar to a natural abundance study than a pulse labeling study and consequently has a different set of requirements in terms of measurement precision and level of background noise. For instance, the soil matrix should be free of fine sand in this case, or chemical microscopy methods like SEM-EDX [44] are required to distinguish garnet particles from the natural fine sand fraction.

Other limitations are posed by the sample size and image resolution. Macropores in natural soil which are induced by desiccation cracks and bioturbation are too big to be captured adequately in 5 ml samples (d:12.5 mm). Our focus is on the small scale dynamics of the inter- and intraaggregate pore space which we believe is highly relevant especially for the turnover of organic matter. This requires a sample size that is small enough to detect the garnet fine sand fraction. A simultaneous investigation of the pore architecture at both scales calls for a hierarchical sampling scheme [19] in which bigger soil cores are scanned first to analyze the macropore network and smaller subsamples are extracted subsequently based on the location in the first scan. The image resolution of 8 *μ*m which was used in this study resulted in a good compromise between visible details and a sufficiently big volume to representatively capture interaggregate pores and garnet particles. Micropores in which soil organic matter is protected against decay are not visible in *μ*CT images. However, we think that micropores are present everywhere all the time. What turns them into oxygen depleted regions is the distance to air-filled mesopores. This distance between garnet powder and mesopores is in fact what we address with our conceptual idea of soil structure turnover.

Finally, garnet particles can be considered as chemically stable for typical timescales of incubation experiments or field experiments. Almandine is a nesosilicate with dissolution rates mainly depending on soil pH, grain size and the existence of protective oxide layers. For instance, for a garnet powder of slightly smaller grain size (d:30±8.3 *μ*m) the reported almandine dissolution rate (weight loss per mineral surface area after 90 days) at pH 3.6 (9.8 mg/m^2^) was two orders of magnitude higher than at pH 8 (0.16 mg/m^2^) [45]. Moreover, the field dissolution of garnet minerals usually amounts to 0.1–10% of dissolution of pure powders, because a high percentage of moisture is stagnant, i.e. ions in solution reach equilibrium which slows down dissolution [46]. Using these values we may assume a dissolution rate of 5 × 10^−7^ mg/(mm^2⋅^ a) for an agricultural soil like that in Bad Lauchstädt with pH 7.3 [47]. A rough calculation shows that the dissolution of a spherical garnet grain from a diameter of d = 50 *μ*m (lower end of the range used in this study) to d = 25 *μ*m (minimum size of detectable objects at 8*μ*m image resolution) takes 10000 years in this case.

## Conclusion

Microscopic changes of soil structure during soil compaction were analyzed with *μ*CT. The increase in bulk density due to compression of soil leads to consistent changes in porosity and mean pore diameter. Pore connectivity in turn remains high initially and breaks down suddenly within a narrow porosity range. We demonstrated that these pore space attributes provide limited insights into structural properties associated with matter turnover. This knowledge gap is closed by analyzing the contact distances between soil and pores and therewith the diffusion lengths for oxygen and nutrients. For the first time we have delineated the conceptual framework and the image processing workflow to study the evolution of contact distances for small garnet particles. Similar to stable isotope labeling of carbon pools, a structure labeling experiment can be devised by bringing garnet particles into contact with aggregate boundaries. In this way, the “pool” of small contact distances to inter-aggregate pores is highly enriched and the dilution into all distance “pools” can be studied over time. In this framework, soil structure turnover manifests itself as an evolution of mean particle contact distances towards the mean contact distance of bulk soil. We have shown that soil compaction entails a large physical displacement of particles due to strong deformation of soil. However, this movement of particles is embedded in the collective movement of all soil constituents. The mean contact distance for particles increases, but not stronger than the contact distance of bulk soil. Yet, particles have not randomized their position relative to pores. This randomization does not depend on the physical extent of particle movement as analyzed with digital image correlation, but on a changing spatial context of particles through formation and destruction of nearby pores. With this conceptual approach the effect of soil compaction on soil structure can be clearly separated from other processes which are expected to occur during soil structure turnover. Finally, the structure labeling approach to quantify soil structure turnover rates can be readily combined with stable isotope labeling of organic compounds to quantify matter turnover.

## Supporting Information

S1 File Image processing workflow. (ZIP)

S2 File Contact distances for regular sphere packings. (ZIP)

## References

[1] Bronick CJ, Lal R. Soil structure and management: a review. Geoderma. 2005;124:3–22. 10.1016/j.geoderma.2004.03.005

[2] Schmidt MWI, Torn MS, Abiven S, Dittmar T, Guggenberger G, Janssens IA, et al Persistence of soil organic matter as an ecosystem property. Nature. 2011;478(7367):49–56. 10.1038/nature10386 21979045

[3] Six J, Bossuyt H, de Gryze S, Denef K. A history of research on the link between (micro)aggregates, soil biota, and soil organic matter dynamics. Soil & Tillage Research. 2004;79:7–31. 10.1016/j.still.2004.03.008

[4] Six J, Elliott E, Paustian K, Doran J. Aggregation and soil organic matter accumulation in cultivated and native grassland soils. Soil Science Society of America Journal. 1998;62(5):1367–1377. 10.2136/sssaj1998.03615995006200050032x

[5] Plante AF, McGill WB. Soil aggregate dynamics and the retention of organic matterin laboratory-incubated soil with differing simulated tillage frequencies. Soil & Tillage Research. 2002;66:79–92. 10.1016/S0167-1987(02)00015-6

[6] von Lützow M, Kögel-Knabner I, Ekschmitt K, Matzner E, Guggenberger G, Marschner B, et al Stabilization of organic matter in temperate soils: mechanisms and their relevance under different soil conditions—a review. European Journal of Soil Science. 2006;57(4):426–445. 10.1111/j.1365-2389.2006.00809.x

[7] Angers DA, Recous S, Aita C. Fate of carbon and nitrogen in water-stable aggregates during decomposition of 13C15N-labelled wheat straw in situ. European Journal of Soil Science. 1997;48(2):295–300. 10.1111/j.1365-2389.1997.tb00549.x

[8] McMahon SK, Williams MA, Bottomley PJ, Myrold DD. Dynamics of microbial communities during decomposition of carbon-13 labeled ryegrass fractions in soil. Soil Science Society of America Journal. 2005;69(4):1238–1247. 10.2136/sssaj2004.0289

[9] Moran KK, Six J, Horwath WR, van Kessel C. Role of mineral-nitrogen in residue decomposition and stable soil organic matter formation. Soil Science Society of America Journal. 2005;69(6):1730–1736. 10.2136/sssaj2004.0301

[10] Puget P, Chenu C, Balesdent J. Dynamics of soil organic matter associated with particle-size fractions of water-stable aggregates. European Journal of Soil Science. 2000;51(4):595–605. 10.1111/j.1365-2389.2000.00353.x

[11] Bernoux M, Cerri CC, Neill C, de Moraes JFL. The use of stable carbon isotopes for estimating soil organic matter turnover rates. Geoderma. 1998;82(1–3):43–58. 10.1016/S0016-7061(97)00096-7

[12] Amelung W, Brodowski S, Sandhage-Hofmann A, Bol R. Chapter 6 Combining Biomarker with Stable Isotope Analyses for Assessing the Transformation and Turnover of Soil Organic Matter vol. 100 of Advances in Agronomy. Academic Press; 2008 p. 155–250. Available from: http://www.sciencedirect.com/science/article/pii/S0065211308006068.

[13] Tisdall JM, Oades JM. Organic matter and water-stable aggregates in soils. European Journal of Soil Science. 1982;33(2):141–163. 10.1111/j.1365-2389.1982.tb01755.x

[14] Christensen BT. Physical fractionation of soil and structural and functional complexity in organic matter turnover. European Journal of Soil Science. 2001;52(3):345–353. 10.1046/j.1365-2389.2001.00417.x

[15] Kuzyakov Y, Friedel JK, Stahr K. Review of mechanisms and quantification of priming effects. Soil Biology and Biochemistry. 2000;32(11–12):1485–1498. 10.1016/S0038-0717(00)00084-5

[16] Mikutta R, Kleber M, Torn MS, Jahn R. Stabilization of Soil Organic Matter: Association with Minerals or Chemical Recalcitrance? Biogeochemistry;77(1):25–56. 10.1007/s10533-005-0712-6

[17] Beare MH, Bruce RR. A comparison of methods for measuring water-stable aggregates: implications for determining environmental effects on soil structure. Geoderma. 1993;56(1–4):87–104. 10.1016/0016-7061(93)90102-Q

[18] Young IM, Crawford JW, Rappoldt C. New methods and models for characterising structural heterogeneity of soil. Soil & Tillage Research. 2001;61:33–45. 10.1016/S0167-1987(01)00188-X

[19] Vogel HJ, Weller U, Schlüter S. Quantification of soil structure based on Minkowski functions. Computers & Geosciences. 2010;36(10):1236–1245. 10.1016/j.cageo.2010.03.007

[20] Nunan N, Ritz K, Rivers M, Feeney DS, Young IM. Investigating microbial micro-habitat structure using X-ray computed tomography. Geoderma. 2006;133(3–4):398–407. 10.1016/j.geoderma.2005.08.004

[21] Kravchenko A, Negassa W, Guber A, Schmidt S. New approach to measure soil particulateorganic matter in intact samples using X-ray computed microtomography. Soil Science Society of America Journal. 2014;78(4):1177–1185. 10.2136/sssaj2014.01.0039

[22] Negassa WC, Guber AK, Kravchenko AN, Marsh TL, Britton H, Rivers ML. Properties of Soil Pore Space Regulate Pathways of Plant Residue Decomposition and Community Structure of Associated Bacteria. PLoS ONE. 2015;10(4):1–22. 10.1371/journal.pone.0123999 PMC4409378 25909444

[23] Jégou D, Brunotte J, Rogasik H, Capowiez Y, Diestel H, Schrader S, et al Impact of soil compaction on earthworm burrow systems using X-ray computed tomography: preliminary study. European Journal of Soil Biology. 2002;38(3–4):329–336.

[24] Schlüter S, Weller U, Vogel HJ. Soil-structure development including seasonal dynamics in a long-term fertilization experiment. Journal of Plant Nutrition and Soil Science. 2011;174(3):395–403. 10.1002/jpln.201000103

[25] Feeney DS, Crawford JW, Daniell T, Hallett PD, Nunan N, Ritz K, et al Three-dimensional Microorganization of the Soil–Root–Microbe System. Microbial Ecology. 2006;52(1):151–158. 10.1007/s00248-006-9062-8 16680511

[26] Crawford JW, Deacon L, Grinev D, Harris JA, Ritz K, Singh BK, et al Microbial diversity affects self-organization of the soil-microbe system with consequences for function. Journal of The Royal Society Interface. 2011; 10.1098/rsif.2011.0679 PMC3350731 22158839

[27] Helliwell JR, Miller AJ, Whalley WR, Mooney SJ, Sturrock CJ. Quantifying the impact of microbes on soil structural development and behaviour in wet soils. Soil Biology and Biochemistry. 2014;74:138–147. 10.1016/j.soilbio.2014.03.009

[28] Hall SA. Discrete and continuum analysis of localised deformation in sand using X-ray *μ*CT and volumetric digital image correlation. Géotechnique. 2010;60:315–322(7).

[29] Peth S, Nellesen J, Fischer G, Horn R. Non-invasive 3D analysis of local soil deformation under mechanical and hydraulic stresses by *μ*CT and digital image correlation. Soil and Tillage Research. 2010;111(1):3–18. 10.1016/j.still.2010.02.007

[30] Schlüter S, Leuther F, Vogler S, Vogel HJ. X-ray microtomography analysis of soil structure deformation caused by centrifugation. Solid Earth. 2016;7(1):129–140. 10.5194/se-7-129-2016

[31] Ketcham RA, Carlson WD. Acquisition, optimization and interpretation of X-ray computed tomographic imagery: applications to the geosciences. Computers & Geosciences. 2001;27(4):381–400. 10.1016/S0098-3004(00)00116-3

[32] Buades A, Coll B, Morel JM. A non-local algorithm for image denoising. In: IEEE Computer Society Conference on Computer Vision and Pattern Recognition, 2005. CVPR 2005. San Diego, CA, 20–25 June 2005. vol. 2; 2005. p. 60–65 vol. 2.

[33] Schlüter S, Sheppard A, Brown K, Wildenschild D. Image processing of multiphase images obtained via X-ray microtomography: A review. Water Resources Research. 2014;50(4):3615–3639. 10.1002/2014WR015256

[34] Gonzalez RG, Woods RG. Digital Image Processing Upper Saddle Creek: Prentice Hall; 2002.

[35] Schlüter S, Weller U, Vogel HJ. Segmentation of X-ray microtomography images of soil using gradient masks. Computers & Geosciences. 2010;36(10):1246–51. 10.1016/j.cageo.2010.02.007

[36] Klein S, Staring M, Murphy K, Viergever MA, Pluim JPW. elastix: A Toolbox for Intensity-Based Medical Image Registration. Medical Imaging, IEEE Transactions on. 2010;29(1):196–205. 10.1109/TMI.2009.2035616 19923044

[37] Doube M, Kłosowski MM, Arganda-Carreras I, Cordelières FP, Dougherty RP, Jackson JS, et al BoneJ: Free and extensible bone image analysis in ImageJ. Bone. 2010;47(6):1076–1079. 10.1016/j.bone.2010.08.023 20817052 PMC3193171

[38] Renard P, Allard D. Connectivity metrics for subsurface flow and transport. Advances in Water Resources. 2013;51(0):168–196. 10.1016/j.advwatres.2011.12.001

[39] Ollion J, Cochennec J, Loll F, Escudé C, Boudier T. TANGO: a generic tool for high-throughput 3D image analysis for studying nuclear organization. Bioinformatics. 2013;29(14):1840–1841. 10.1093/bioinformatics/btt276 23681123 PMC3702251

[40] Ohser J, Mücklich P. Statistical Analyisis of Microstructures in Material Science. New York: Wiley & Sons; 2000.

[41] Ebrahimi A, Or D. Hydration and diffusion processes shape microbial community organization and function in model soil aggregates. Water Resources Research. 2015;51(12):9804–9827. 10.1002/2015WR017565

[42] Hazlett RD. Simulation of capillary-dominated displacements in microtomographic images of reservoir rocks. Transport in Porous Media. 1995;20(1–2):21–35. 10.1007/BF00616924

[43] Kumahor S, de Rooij G, Schlüter S, Vogel HJ. Water Flow and Solute Transport in Unsaturated Sand—A Comprehensive Experimental Approach. Vadose Zone Journal. 2015;14 10.2136/vzj2014.08.0105

[44] Hapca S, C BP, Clare W, Murray LR, Wilfred O. Three-Dimensional Mapping of Soil Chemical Characteristics at Micrometric Scale by Combining 2D SEM-EDX Data and 3D X-Ray CT Images. PLoS ONE. 2015;10(9):e0137205 10.1371/journal.pone.0137205 26372473 PMC4570663

[45] Nickel E. Experimental dissolution of light and heavy minerals in comparison with weathering and intrastratal solution. Contributions to Sedimentology. 1973;1:1–68.

[46] Velbel MA. Constancy of silicate-mineral weathering-rate ratios between natural and experimental weathering: implications for hydrologic control of differences in absolute rates. Chemical Geology. 1993;105(1–3):89–99. 10.1016/0009-2541(93)90120-8

[47] Altermann M, Rinklebe J, Merbach I, Körschens M, Langer U, Hofmann B. Chernozem—Soil of the Year 2005. Journal of Plant Nutrition and Soil Science. 2005;168:725–740. 10.1002/jpln.200521814

